# Causal Relationship Between Gut Microbiota, Blood Metabolites, and Intervertebral Disc Degeneration: A Two‐Step, Two‐Sample Bidirectional Mendelian Randomization Study

**DOI:** 10.1002/jsp2.70078

**Published:** 2025-05-29

**Authors:** Yi‐Ping Zheng, Dong‐Lin Yang, Lu‐Yang Wang, Xi‐Zhong Zhu, Xing‐Chen Li, Jin‐Hong Miao, Yu‐Sheng Xu

**Affiliations:** ^1^ Department of Orthopaedic The First Affiliated Hospital of Zhengzhou University Zhengzhou China; ^2^ Department of Quality Control The First Affiliated Hospital of Zhengzhou University Zhengzhou China

**Keywords:** blood metabolites, gut microbiota, intervertebral disc degeneration, Mendelian randomization

## Abstract

**Background:**

Some studies have shown that gut microbiota may be associated with intervertebral disc degeneration. However, the causal effects between gut microbiota and IVDD and whether blood metabolites act as a mediator remain unclear. The objective of this study was to investigate the causal relationship between gut microbiota and intervertebral disc herniation, with a focus on the potential mediating role of blood metabolites.

**Methods:**

Gut microbiota, blood metabolites, and IVDD data were identified from large‐scale genome‐wide association studies (GWAS) summary data. Then we used Mendelian randomization analysis to investigate the causal relationships between gut microbiota, blood metabolites, and intervertebral disc degeneration, using the inverse variance‐weighted method as the primary outcome measure. Subsequently, we conducted sensitivity analyses to ascertain the robustness of the results by testing for heterogeneity and horizontal pleiotropy. In addition, we explored blood metabolites as a mediating factor in the pathway from gut microbiota to IVDD.

**Results:**

We identified 6 taxa that were strongly associated with the incidence of intervertebral disc herniation. There were 8 positive and 13 negative causal effects between genetic liability in the blood metabolites and IVDD. The mediation analysis revealed that the connections among genus Comamonas B, family Halomonadaceae, family UBA6960, and IVDD were mediated by ADP to glycine ratio, 1,3‐dimethylurate levels, 3‐hydroxy‐2‐methylpyridine sulfate levels, and Histidine levels. Each of these accounted for 7.77%, 9.04%, 12.56%, and 11.76%, respectively.

**Conclusions:**

Our study provides evidence supporting a potential causal relationship between certain microbial taxa and intervertebral disc degeneration. This study focuses on the mediation of specific blood metabolites, which suggests that they may represent potential targets for intervention.

## Graphical abstract

We have identified several specific gut microbiota that may have an effect on intervertebral disc degeneration, and have found possible blood metabolites that mediate the effects of the gut microbiota on intervertebral disc degeneration. This study provides new insights into the mechanisms by which gut microbes are involved in the development of intervertebral disc disease and lays the foundation for further basic experiments.

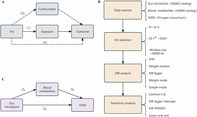

## 1 Introduction

Low back pain (LBP) is one of the most prevalent disorders worldwide, with a significant impact on the financial and social costs. The prevalence of low back pain is on the rise globally, year on year [1]. Neuropathic pain caused by intervertebral disc degeneration (IVDD) has become a growing concern, particularly as aging populations and sedentary lifestyles exacerbate spinal health challenges [2]. Intervertebral disc is a complex structure comprising three components: the nucleus pulposus (NP), the annulus fibrosus (AF), and the cartilaginous endplates (CEP), which are situated between adjacent vertebral bodies [3]. Intervertebral disc degeneration (IVDD) refers to the degeneration of disc structure and function, driven by multifactorial mechanisms such as aging, mechanical stress, genetic predisposition, obesity, smoking and diabetes [4]. There is evidence that environmental and pharmacological factors (such as the use of antibiotics) may further regulate the progression of LBP. Long‐term antibiotic exposure is related to Gut microbiota disorder [5]. Current treatments for disc degeneration include the administration of painkillers, anti‐inflammatory drug therapy, physiotherapy, and surgical intervention [6]. In recent years, new strategies such as stem cell, gene, and small molecule therapies have emerged [7]. However, the majority of these approaches focus on the alleviation of symptoms rather than the treatment of the underlying disease process. Consequently, there is an urgent need to identify new therapeutic targets and strategies, particularly from the perspective of early prevention and intervention.

The growing body of evidence suggests that gut microbiota (GM) plays an important role in IVDD. Gut microbiota (GM) refers to the totality of microorganisms that reside within the human gut, including bacteria, fungi, and protozoa, which have the potential to influence the host's metabolic, immune, and endocrine environments through a range of mechanisms [8, 9]. A consensus is emerging that IVDD is closely related to GM. Propionibacterium may be the first bacterium found to be associated with IVDD [10]. The concept of the ‘gut‐spine axis’ emphasizes the possible interaction between GM and spinal disorders [11]. Clinical and animal studies suggest that GM may influence IVDD by regulating blood metabolites such as short‐chain fatty acids [12].

In the majority of observational studies, it is relatively easy to bias the association between GM and IVDD as a result of interference from confounding factors, which may compromise the reliability of the findings. Mendelian randomization (MR) is an epidemiological research method that employs SNPs as instrumental variables (IVs) to investigate the causal relationship between an exposure and disease. This method is founded upon the principles of Mendel's genetic law, which serves to minimize the impact of confounding factors [13]. Mediation Mendelian Randomization is an extension of the MR approach, whereby the impact of an exposure factor on outcomes is investigated through the lens of one or more mediating variables [14]. The aim of this study was to analyze the causal relationship between GM, blood metabolites, and IVDD using two‐sample bidirectional MR and mediation MR to identify potential metabolites and provide strategies for early diagnosis and clinical management.

## 2 Methods

### 2.1 Study Design

**FIGURE 1:**
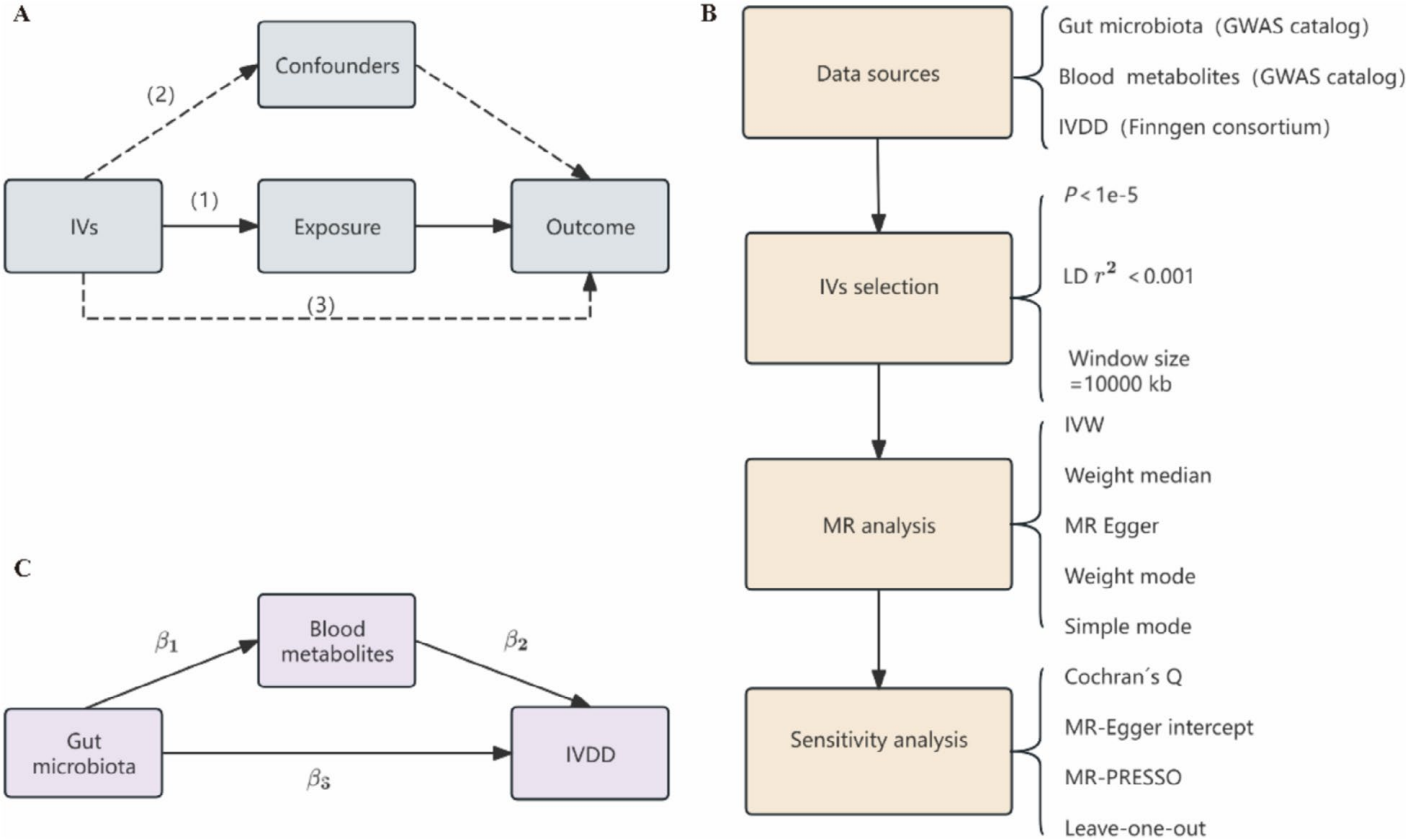
(A) Three main assumptions of Mendelian randomization. (1) relevance assumption, (2) independence assumption, (3) exclusion restriction assumption. (B) The flowchart of a MR study. (C) Two‐step MR study of GM on IVDD mediated by blood metabolites. IVDD, intervertebral disc herniation; IVs, instrumental variables; IVs, instrumental variables; IVW, inverse variance weighted; LD, linkage disequilibrium; *β*_1_, influence of GM on metabolites; *β*_2_, influence of metabolites on IVDD; *β*_3_, total effect GM on IVDD.

As we all know, the independence, relevance and exclusion restriction assumption have been the three central assumptions of MR [15]. Based on these, single nucleotide polymorphisms (SNPs), a genetic variation trait, were employed as instrumental variables following a process of selection based on their suitability. A two‐sample MR analysis was employed to screen and identify GM from 473 taxa and metabolites from 1400 blood metabolites that were significantly causally associated with IVDD. Moreover, to ensure the directionality and robustness of the results, we used bidirectional MR randomization and sensitivity analyses. Further, we used a two‐step MR analysis approach aimed at exploring the potential role of metabolites in mediating the association between GM and IVDD. Mediation MR can infer causality more accurately and overcome confounding and reverse causality problems common in traditional observational studies through genetic information. The study comprises three principal components as shown in Figure 1. This research follows the Strengthening STROBE‐MR Statement as well [16].

### 2.2 Data Sources

The data of GM came from a single population cohort study. Complete summary statistics of microbial taxa with genome‐wide significant hits were publicly available in the NHGRI‐EBI GWAS Catalog (https://www.ebi.ac.uk/gwas/) from accession GCST90032172 to GCST90032644 [17]. The 473 bacterial traits can be classified into 17 phylum, 17 class, 19 order, 77 family, 156 genus and 187 species. This research characterized genetic variations associated with microbial abundances in a single population‐based cohort of 5959 genotyped individuals with matched gut microbial metagenomes. These data cover a richer and more comprehensive range of GM than previous studies of the same type [18, 19].

The present study utilized blood metabolites data from a genome‐wide association study conducted as part of the Canadian Longitudinal Study of Aging (CLSA), analyzing 1091 metabolites and 309 metabolite ratios in 8299 participants [20]. The metabolites can be divided into eight main categories: lipid, amino acid, xenobiotics, nucleotide, cofactor and vitamins, carbohydrate, peptide, and energy. We can also obtain the data from GWAS Catalog, numbers ECST90199621‐ECST90201020.

The GWAS summary data of IVDD were derived from the Eleventh version of the Finngen consortium (https://r11.finngen.fi/), including 46 205 cases and 322 314 controls [21]. The main diagnostic criteria used for the disease was defined in the ICD‐10—M51, ICD‐9—722, and ICD‐8—725, excluded ICD‐9—7220|7224|7227|7228A, ICD‐8—7250. No additional ethical approval or consent was required as this study was based on publicly available data. Detailed information on these studies is provided in Table 1.

**TABLE 1:** Summary of GWAS datasets included in this research.

Phenotype	Data source	Participants	Ancestry	Year
Gut microbiota	GWAS catalog	5959	Finn	2023
Blood metabolites	GWAS catalog	8299	European	2023
IVDD	FinnGen	368 519 (46 205 cases/322 314 controls)	Finn	2024

### 2.3 Selection of Instrumental Variables

We selected the SNPs that were significantly associated with each of the GMs and blood metabolites as instrumental variables (IVs). However, due to the limited number of IVs obtained with a strict threshold (*p* < 5 × 10^−8^), we relaxed the significance threshold to be *p* < 1.0 × 10^−5^. We set the window size > 10 000 Kb, *r* ^2^ < 0.001 to ensure the independence of the IVs and to reduce the effects of linkage disequilibrium (LD) [22]. The harmonization process was employed to eliminate palindromic and incompatible alleles, thereby ensuring the quality and accuracy of the data. Finally, the weak IVs were excluded through the calculation of the F statistic for each SNP. In accordance with the findings of previous research, an F‐statistic value exceeding 10 was deemed that can avoid bias from weak instruments [23, 24]. The formula is as follows: *F* = *R* ^2^ (*n*‐*k*‐1)/*k*(1‐*R* ^2^), *R* ^2^ = 2 × EAF×(1 − EAF) × *β* ^2^. *n* represents the sample size, while *k* is the number of IVs. All instrumental variables (IVs) were screened against SNPs associated with known confounders (e.g., BMI, smoking behavior) using the PhenoScanner database [25]. SNPs with pleiotropic effects (*p* < 1 × 10^−5^) were excluded.

### 2.4 Statistical Analysis

Firstly, we conducted a screening process to identify IVs that were strongly associated (*F* > 10) with 473 gut microbiota and 1400 blood metabolites. Subsequently, we performed two‐sample bidirectional MR analyses with IVDD. A variety of methods were applied, including inverse variance weighted (IVW), MR Egger, and weighted median. The IVW method was selected as the primary outcome. This is because it provides the most reliable estimate of the effect [26]. The MR Egger and weighted median methods were employed as secondary methods to validate and screen the results. The results of the MR were expressed as odds ratios (ORs) with the corresponding 95% confidence intervals (CI). When the *p*‐value for IVW was less than 0.05 and when IVW was in the same direction as MR‐Egger, this result was statistically significant [27]. We then performed sensitivity analyses to assess heterogeneity among genetic variants using Cochran's *Q* test [28], where the intercept of the MR‐Egger regression [29] was used to detect horizontal pleiotropy and the slope indicates the causal estimate adjusted for pleiotropy. Leave‐one‐out analysis and MR‐Pleiotropy Residual Sum and Outlier (MR‐PRESSO) methods were used to assess the robustness of the results obtained and whether outlying SNPs affected causality [30, 31]. Leave‐one‐out analysis was conducted by sequentially removing each SNP and re‐running the Mendelian Randomization analysis. This approach helps determine if any single SNP has a disproportionate influence on the overall causal effect and provides insight into whether the results are robust to the exclusion of individual SNPs. MR‐PRESSO method identifies potential outlying SNPs that may distort the causal estimates. After detecting outliers, MR‐PRESSO performs a correction to remove their effects, ensuring that the causal relationship remains unbiased.

GM and blood metabolites with significant causal effects on IVDD were included after the exclusion of GM and metabolites with reverse causality to the outcome. Subsequently, using the aforementioned identified metabolites as outcome and the GM as exposures, we investigated whether there was a causal effect of the GM on blood metabolites. If this is the case, a multiple MR analysis will be performed to ascertain whether the metabolites are mediators in the pathway from GM to IVDD.

The study was statistically analyzed using the TwoSampleMR package (version 0.6.4) in R (version 4.2.1). A *p*‐value of less than 0.05 was considered statistically significant.

## 3 Results

### 3.1 MR Analysis for Gut Microbiota and IVDD

**FIGURE 2:**
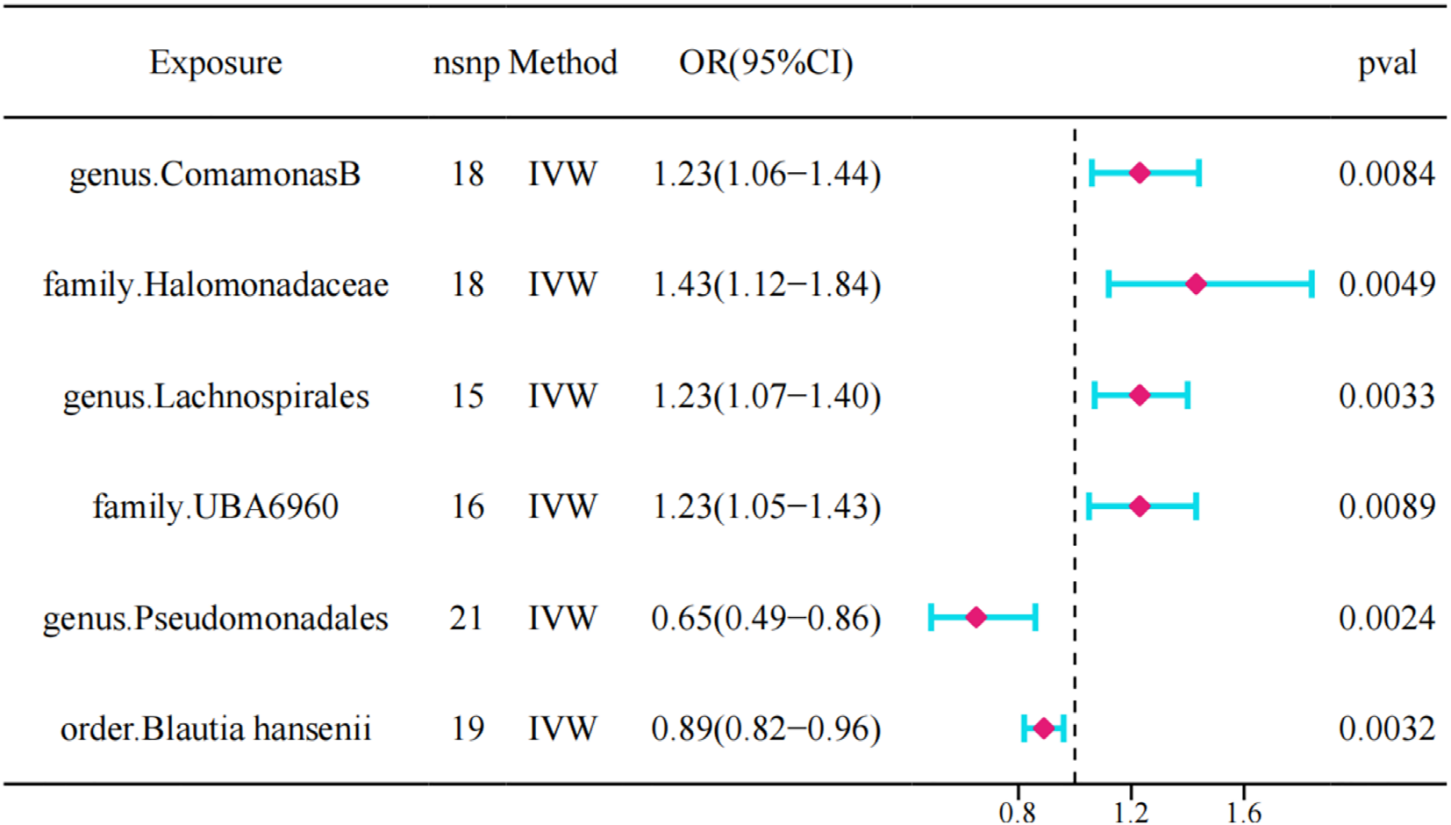
Forest plot of MR analysis between six gut microbiota and IVDD.

In the MR. analysis, F statistic was greater than 10 for each of the SNPs included. We screened six gut microbiota that were significantly associated with an increased or decreased risk of IVDD, and detailed information on the SNPs for the six gut microbiota can be found in Supporting Information S1. As shown in Figure 2, we found that high abundance of genus. Comamonas B (OR = 1.23 95% CI: 1.05–1.44, *p* = 0.008), family. Halomonadaceae (OR = 1.43 95% CI: 1.11–1.84, *p* = 0.004), genus. Lachnospirales (OR = 1.22 95% CI = 1.06–1.40, *p* = 0.003) and family. UBA6960 (OR = 1.22 95% CI: 1.05–1.43, *p* = 0.008) are a potential risk factor for IVDD and may have a role in the development of IVDD. Conversely, genus. Pseudomonadales (OR = 0.65 95% CI = 0.49–0.86, *p* = 0.002) and order. *Blautia hansenii* (OR = 0.89 95% CI = 0.82–0.96, *p* = 0.003) may be protective factors for IVDD. Meanwhile, the reverse MR analysis demonstrated that there was no notable causal correlation between IVDD and any of the six taxa when IVDD was employed as an exposure factor. The Cochran's *Q* test and MR‐Egger intercept test yielded no evidence of heterogeneity or horizontal pleiotropy in this MR analysis (*p* > 0.05). The leave‐one‐out and MR‐PRESSO test analysis also demonstrated the absence of any abnormal SNPs. These results demonstrate the validity and robustness of our findings. The scatterplot illustrates the causal relationship between the six intestinal microbiota and IVDD (Supporting Information S2). In this process, we ensured consistency in the direction of the results of the five different analyses, although not all of them met the criteria for statistical significance.

### 3.2 MR Analysis for Blood Metabolites and IVDD

**FIGURE 3:**
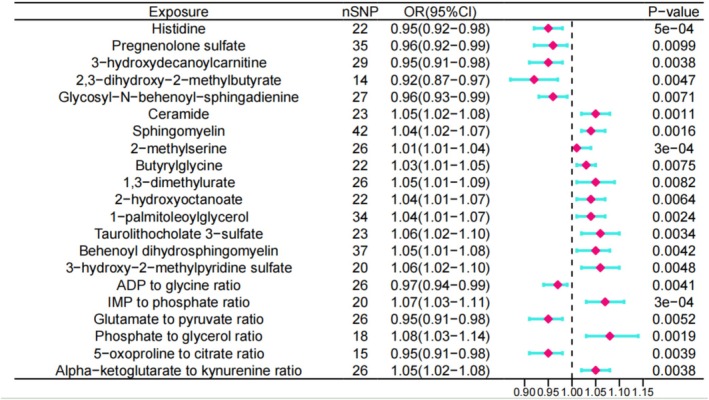
Forest plot of MR analysis between 21 blood metabolites and IVDD.

We performed inverse variance weighted (IVW) analyses and excluded metabolites with significant heterogeneity, pleiotropic characteristics, and potential reverse causality with IVDD. Finally, we screened for 21 key metabolites (6 metabolite ratio and 15 metabolites) that may have a causal effect on IVDD (Supporting Information S3). The results are shown below. 3‐hydroxydecanoylcarnitine (OR = 0.94 95% CI = 0.91–0.98, *p* = 0.0037), Histidine levels (OR = 0.95 95% CI = 0.92–0.98, *p* = 0.0005), 2,3‐dihydroxy‐2‐methylbutyrate (OR = 0.91 95% CI = 0.86–0.97, *p* = 0.0046), Glycosyl‐N‐behenoyl‐sphingadienine (OR = 0.95 95% CI = 0.92–0.98, *p* = 0.0071) and Pregnenolone sulfate levels (OR = 0.95 95% CI = 0.92–0.98, *p* = 0.0099) had a protective causal effect on IVDD. Conversely, 2‐methylserine (OR = 1.02 95% CI = 1.01–1.04, *p* = 0.00033), Ceramide (OR = 1.05 95% CI = 1.01–1.08 *p* = 0.001), Sphingomyelin (OR = 1.04 95% CI = 1.01–1.06, *p* = 0.0015), 1‐palmitoleoylglycerol (OR = 1.04 95% CI = 1.01–1.07, *p* = 0.0023), Taurolithocholate 3‐sulfate (OR = 1.05 95% CI = 1.01–1.09, *p* = 0.0033), Behenoyl dihydrosphingomyelin (OR = 1.04 95% CI = 1.01–1.08, *p* = 0.0042), 3‐hydroxy‐2‐methylpyridine sulfate (OR = 1.05 95% CI = 1.01–1.10, *p* = 0.0047), 2‐hydroxyoctanoate (OR = 1.03 95% CI = 1.01–1.06, *p* = 0.0063), Butyrylglycine (OR = 1.02 95% CI = 1.007–1.05, *p* = 0.0075) and 1,3‐dimethylurate levels (OR = 1.04 95% CI = 1.01–1.08, *p* = 0.0081) were risk factors for IVDD (Figure 3). In addition, the sensitivity analyses did not show any anomalous results (Supporting Information S4). Therefore, these 21 identified blood metabolites were deemed suitable for further analysis.

### 3.3 MR Analysis for Gut Microbiota and Blood Metabolites

In this study, we found that GM and blood metabolites have a direct causal effect on IVDD. Further, blood metabolites appear to play a bridging role, mediating the influence of the GM on IVDD. Notably, an important prerequisite for this mediating effect is that there is a significant correlation between the GM and blood metabolites. Therefore, in order to further investigate whether GM can influence IVDD through the blood metabolic pathway, we performed MR analyses between 6 identified gut microbiota and 21 blood metabolites and metabolite ratios causally associated with IVDD.

**FIGURE 4:**
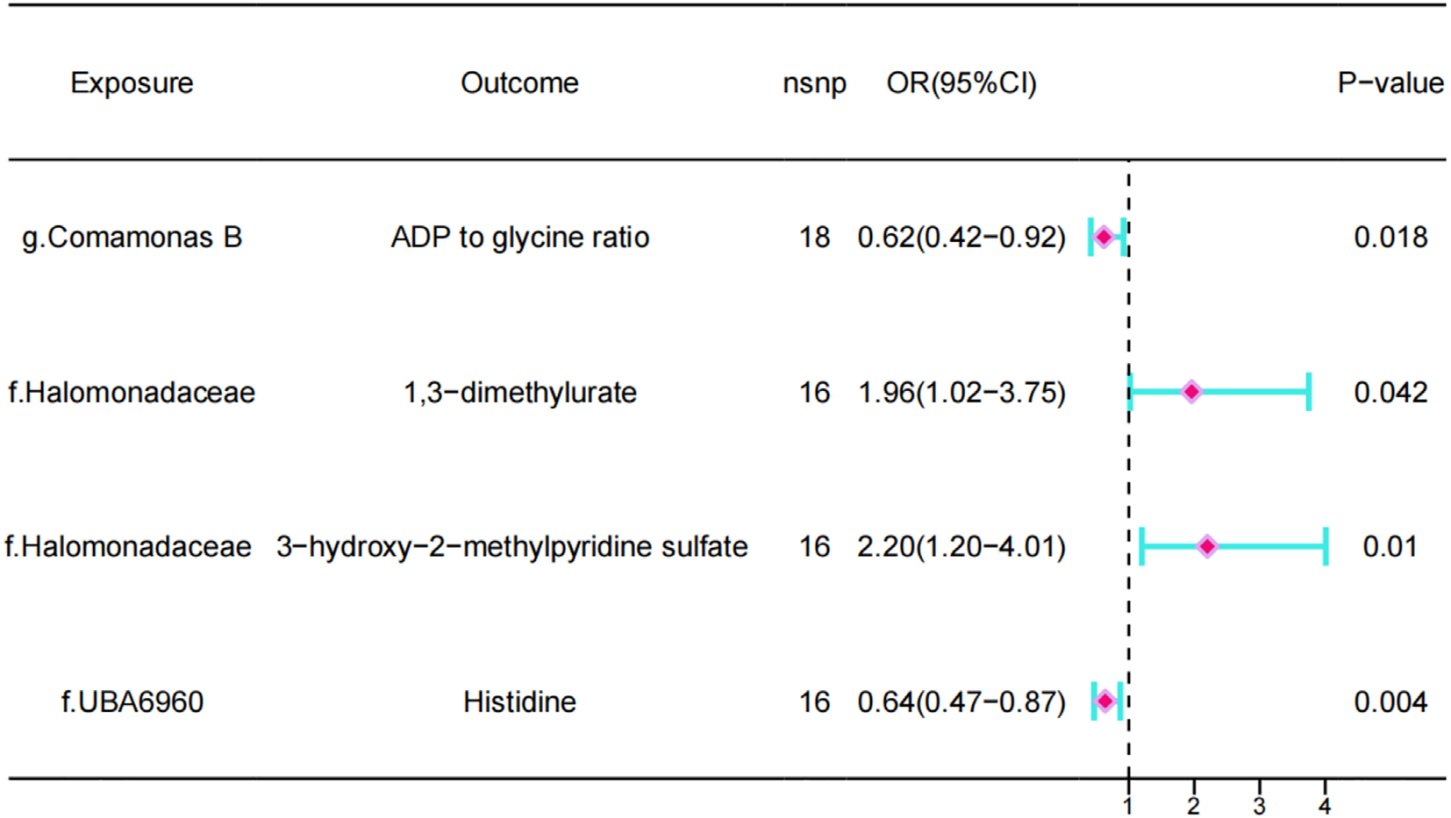
Forest plot of MR analysis between 6 gut microbiota and 21 blood metabolites.

The IVW method shows a causal effect between genus. Comamonas and ADP to glycine ratio (OR = 0.62 95% CI = 0.41–0.92, *p* = 0.018), family. Halomonadaceae and 3‐hydroxy‐2‐methylpyridine sulfate levels (OR = 2.19 95% CI = 1.20–4.01, *p* = 0.01), family. Halomonadaceae and 1,3‐dimethylurate levels (OR = 1.96 95% CI = 1.02–3.75, *p* = 0.04), family. UBA6960 and Histidine levels (OR = 0.63 95% CI = 0.47–0.87, *p* = 0.004) (Figure 4, with detailed information in Supporting Information S5).

### 3.4 Mediation Analysis

On the basis of the appeal results, mediation analysis was used to investigate the role of metabolites in the mediation of the association. The results showed that in the mediation MR analysis, G. Comamonas B played a mediating role through the ADP to glycine ratio, and this mediation pathway accounted for 7.78% of the total effect. Family, Halomonadaceae played a mediating role through 1,3‐dimethylurate levels, which accounted for 9.04% of its total association with IVDD, and similarly by 3‐hydroxy‐2‐methylpyridine sulfate levels, which accounted for 12.56% (Table 2).

**TABLE 2:** The proportion of the effect of metabolites in the causal relationship between gut microbiota and disc degeneration.

Exposure	Mediator	Outcome	*β*_all_	*β*_indir_	*β*_dir_	Proportion
g.Comamonas B	ADP to glycine ratio	IVDD	0.2093615	0.01629125	0.1930703	7.78%
f.Halomonadaceae	1,3‐dimethylurate levels	IVDD	0.3602667	0.03257683	0.3276899	9.04%
f.Halomonadaceae	3‐hydroxy‐2‐methylpyridine sulfate levels	IVDD	0.3602667	0.04525938	0.3150073	12.56%
f.UBA6960	Histidine levels	IVDD	0.205047	0.02412032	0.1809267	11.76%

Abbreviations: *β*_all_: total effect GM on IVDD; *β*_dir_: direct effect (*β*_dir_ = *β*_all_–*β*_1_ × *β*_2_); *β*_indir_: indirect effect = *β*_1_(Influence of GM on metabolites) × *β*_2_ (Influence of metabolites on IVDD).

## 4 Discussion

The gut microbiota plays a critical role in the pathogenesis of IVDD. Our analysis, based on the most recent publicly available GWAS summary statistics, incorporates data from 473 species of gut microbiota and 1400 species of metabolites, making it a more comprehensive and detailed analysis. According to our study, we have revealed that six taxa and 21 blood metabolic pathways were involved in the occurrence and development of IVDD, after correction by weighted median and MR‐PRESSO assessments. The mediation analysis showed that the connections among genus Comamonas B, family Halomonadaceae, family UBA6960 and IVDD were mediated by ADP to glycine ratio, 1,3‐dimethylurate levels, 3‐hydroxy‐2‐methylpyridine sulfate levels and Histidine levels. Each of these accounted for 7.77%, 9.04%, 12.56%, and 11.76%, respectively.

GM serves as the second brain of the organism and plays an important role in regulating various aspects of the organism's internal environmental homeostasis and immune defense system, and so forth [32]. Previously, it was assumed that intervertebral discs were sterile. However, Rajasekaran et al. conducted 16SrRNA sequencing of the genomic DNA of the lumbar intervertebral disc, revealing the presence of a microbiome in human intervertebral disc [33]. Subsequently, the concept of the “gut‐disc” axis was introduced and began to attract attention [11, 12]. In this study, genus. Comamonas, family. Halomonadaceae and family. UBA6960 was concluded to be a risk factor for IVDD. Conversely, genus. Pseudomonadales and order. *Blautia hansenii* may be protective factors for IVDD. Genus. Comamonas are a group of gram‐negative bacteria. It was demonstrated that the genus Comamonas, an opportunistic pathogen, exhibits a strong correlation with obesity, diabetes, anorexia nervosa, and other diseases [34, 35]. The abundance of genus. Comamonas was significantly increased in the intestines of mice fed a high‐fat diet, suggesting that this microbiota is closely associated with lipid or energy metabolism. As we all know, obesity and diabetes are identified as risk factors for disc degeneration [36]. Therefore, we hypothesized that there could be a potential association between genus. Comamonas and disc degeneration. Our study also confirmed the significant association between them and provided genetic evidence for future study. Additionally, The study found that family. Halomonadaceae and family. UBA6960 were risk factor for GBM. Wang et al. reported similar results [37]. However, the function of these bacterium in the organism is poorly understood, and there are no studies on the association with IVDD. Therefore, further studies are required to clarify the mechanisms of these bacterial taxon in the development of IVDD.

GM can influence the regulation of nutrients absorption and the formation of metabolites by the intestinal epithelium. It has also been shown that GM can diffuse into the disc with blood flow, which in turn affects the metabolism of the disc [38, 39]. The integrity of the epithelial barrier is considered one of the main mechanisms of action of the “gut‐disc axis” [12]. Studies have demonstrated that the supply of nutrients to the intervertebral disc is predominantly dependent on the diffusion of nutrients from peripheral blood vessels. Blood metabolites exert a direct influence on the survival and functionality of disc cells. Therefore, the gut microbiota may indirectly affect disc health by regulating the concentration of metabolites in the bloodstream [40]. Our findings indicate that Comamonas may contribute to the development of IVDD by reducing the ADP to glycine ratio. Additionally, a member of the family. UBA6960 may also play a role in this process by lowering histidine levels. Histidine, a dietary essential amino acid, plays an important role in the scavenging of reactive oxygen and nitrogen species, erythropoiesis and the histaminergic system [41]. As a physiological antioxidant, it can prevent cell death and myelin loss in vivo [42]. In an imaging and metabolomics study, it was observed that the amino acid metabolism axis has the potential to delay disc degeneration through antioxidant effects [43]. Based on the findings of this study, we conclude that UBA6960 reduces histidine levels and decreases the antioxidant capacity of cells in the intervertebral disc, thereby promoting intervertebral degeneration. In contrast, family. Halomonadaceae may have a potentially dangerous effect on IVVD by increasing 3‐hydroxy‐2‐methylpyridine sulfate and 1,3‐dimethylurate levels. 3‐Hydroxy‐2‐methylpyridine sulfate is a substance that is metabolized in the body by the liver and is associated with the metabolism of vitamin B6 [44]. 1,3‐dimethylurate is a product of purine metabolism, which is associated with uric acid metabolism and may play a role in regulating acid–base balance and antioxidant responses in the body. The exact relationship and mediating role of this factor may offer valuable insights into potential therapeutic options for targeting gut microbiota, with the aim of slowing down the development of IVDD. We need further research to explain the mechanism of action of 3‐Hydroxy‐2‐methylpyridine sulfate and 1,3‐dimethylurate in the organism.

Similar to previous studies, we also have demonstrated that gut microbiota may also have an effect on intervertebral disc homeostasis through the regulation of lipid metabolism ratio [45]. This study also supports the idea that intestinal microbiota may influence disc degeneration by influencing the ratio of different lipids, such as Inosine 5′‐monophosphate (IMP) to phosphate ratio, Phosphate to glycerol ratio, Cholesteryl esters to total lipids ratio in medium VLDL and Free cholesterol to total lipids ratio in very large HDL and so forth. However, most gut microbiota can influence several lipids, or one lipid is influenced by several gut microbiota. Thus further research may be needed to establish the specific relationships.

Nonetheless, there are limitations to our study. Firstly, when screening for SNPs in the gut microbiota and metabolites whose data could be used for subsequent analyses, we relaxed the genome‐wide significance threshold (*p* < 1 × 10^−5^) for screening in order to obtain a sufficient number of SNPs, which may affect the reliability of the results. Moreover, although we removed some of the confounding factors such as (e.g., obesity, diabetes), we were unable to remove all of them due to methodological limitations. Secondly, We focused that our study was conducted with a focus on individuals of European ancestry, in order to minimize the potential for bias resulting from population stratification. However, this restricted the extent to which the findings can be applied more widely. Thirdly, it was possible that there may be sample overlap between the data of gut microbiota and IVDD, given that both originate from the FinnGen database. In future studies, further experimental investigation is required to elucidate the role of the gut microbiota in influencing IVDD and the underlying molecular mechanisms.

In conclusion, our study identified 6 microbial taxa and 21 metabolites (included metabolite ratios) that are strongly associated with the development of disc herniation. We also found 4 possible metabolite pathways of 3 gut microbiota that are closely related to disc degeneration. Our MR analyses provided genetic evidence for a potential mediating role of blood metabolites in the causal relationship between the gut microbiota and IVDD.

## Author Contributions

Yi‐Ping Zheng wrote the body of the manuscript and designed the study as well as analyzed the data. Dong‐Lin Yang, Lu‐Yang Wang, and Xi‐Zhong Zhu participated in data collection and article writing. Xing‐Chen Li, Jin‐Hong Miao, and Yu‐Sheng Xu were involved in revising the content and format of the article.

## Conflicts of Interest

The authors declare no conflicts of interest.

## Supporting information


Data S1.

Data S2.

Data S3.

Data S4.

Data S5.
