# Supplementary material for: Causal Relationship Between Gut Microbiota, Blood Metabolites, and Intervertebral Disc Degeneration: A Two‐Step, Two‐Sample Bidirectional Mendelian Randomization Study
Source: JOR Spine. 2025 May 29;8(2):e70078. doi: 10.1002/jsp2.70078 (PMC12120259; doi:10.1002/jsp2.70078)

Supplementary file2 Scatter plot of the relationship between 6 bacterial flora and IVDD using five methods:IVW,weighted median,MR-Egger,weighted mode,and simple mode.


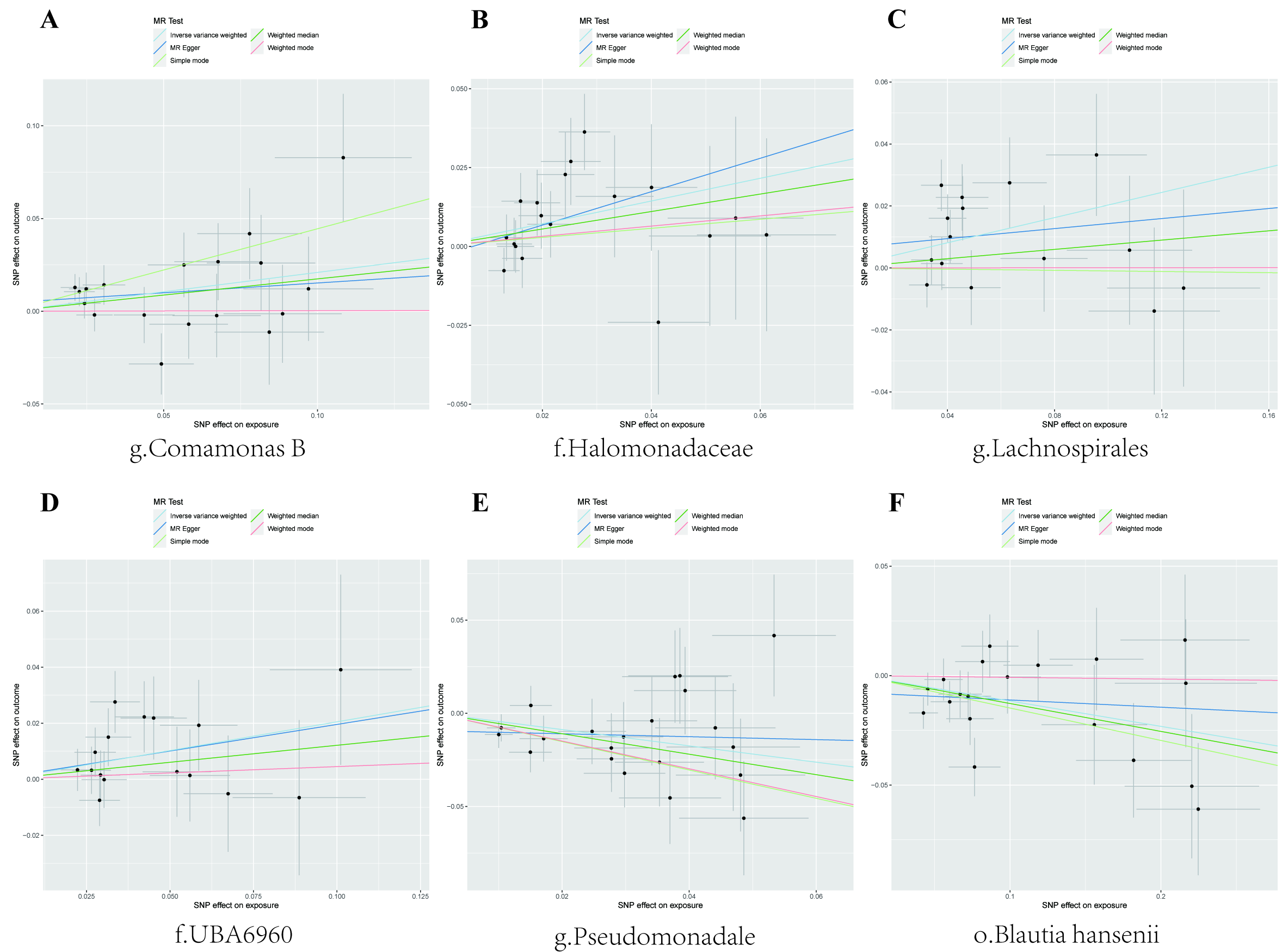

Supplement: Supplementary file 2 — Data S2. [file JSP2-8-e70078-s005.docx]
