# Supplementary material for: Causal Relationship Between Gut Microbiota, Blood Metabolites, and Intervertebral Disc Degeneration: A Two‐Step, Two‐Sample Bidirectional Mendelian Randomization Study
Source: JOR Spine. 2025 May 29;8(2):e70078. doi: 10.1002/jsp2.70078 (PMC12120259; doi:10.1002/jsp2.70078)
Supplement: Supplementary file 4 — Data S4. [file JSP2-8-e70078-s002.docx]

The leave-one-out plot of Mendelian randomization analyses between 21 blood metabolites and IVDD.

| 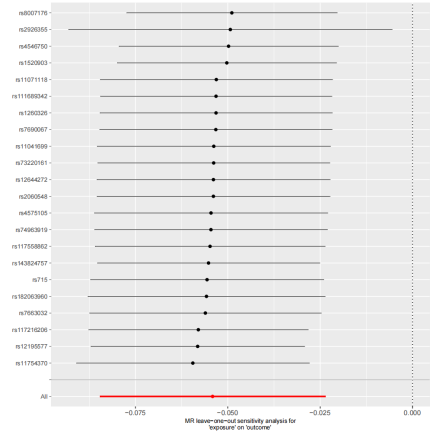 | 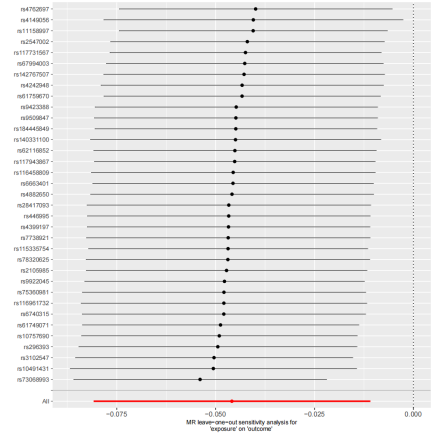 | 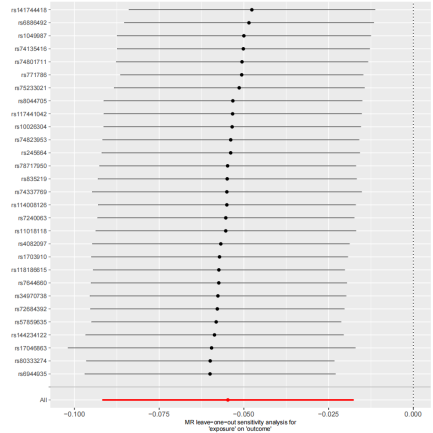 |
| --- | --- | --- |
| Histidine | Pregnenolone sulfate | 3-hydroxydecanoylcarnitine |
| 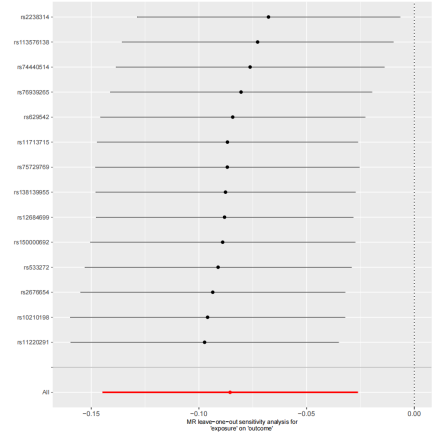 | 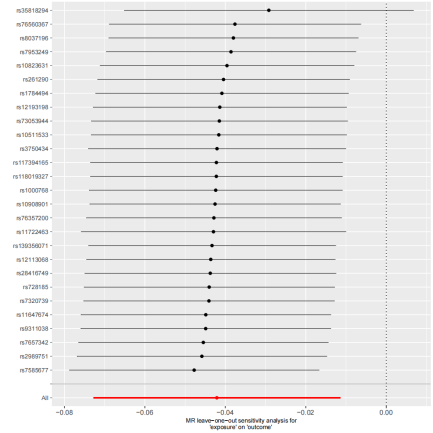 | 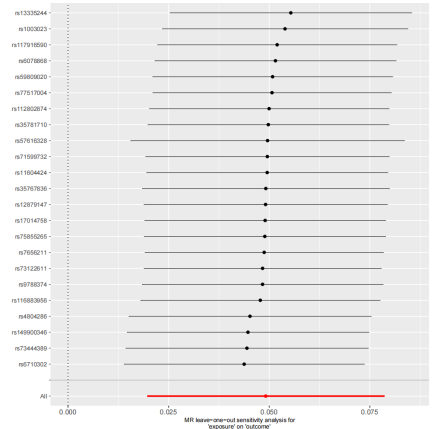 |
| 2,3-dihydroxy-2-methylbutyrate | Glycosyl-N-behenoyl-sphingadienine | Ceramide |
| 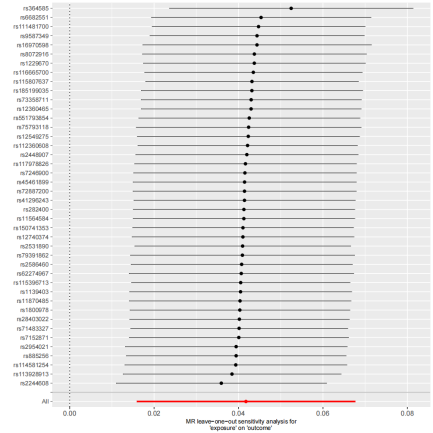 | 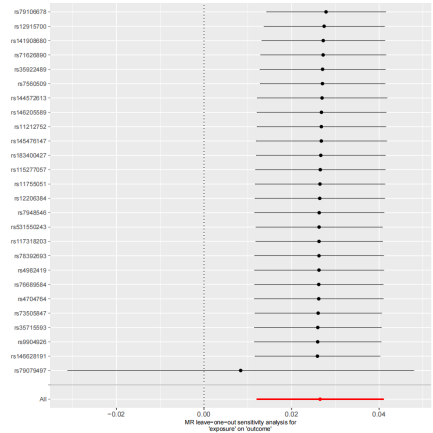 | 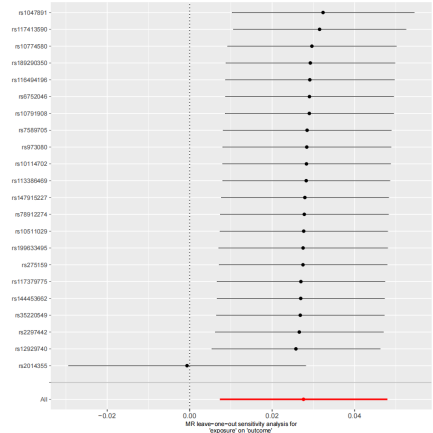 |
| Sphingomyelin levels | 2-methylserine levels | Butyrylglycine levels |
| 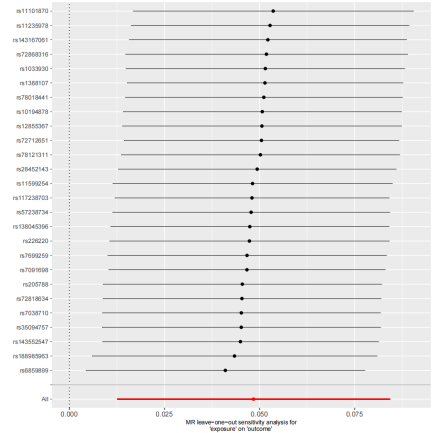 | 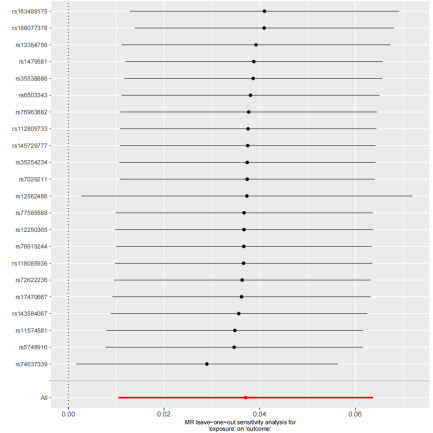 | 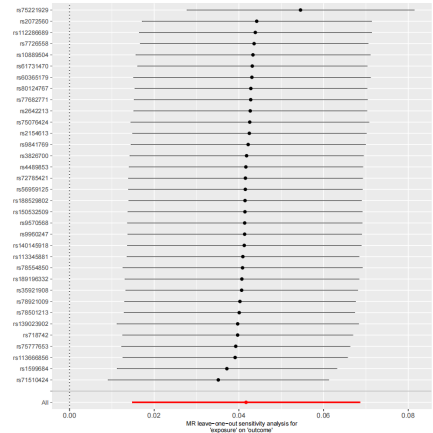 |
| 1,3-dimethylurate levels | 2-hydroxyoctanoate | 1-palmitoleoylglycerol |
| 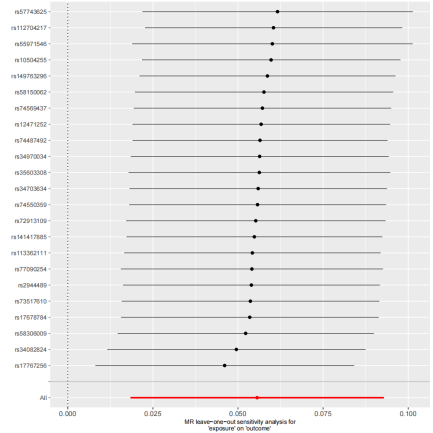 | 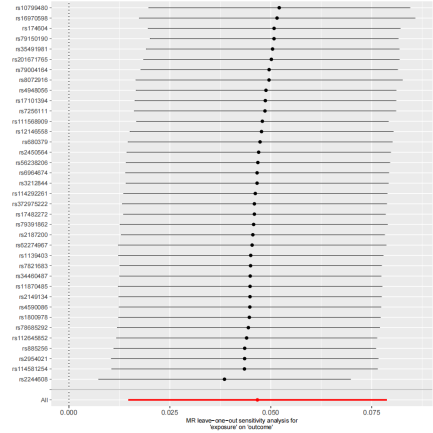 | 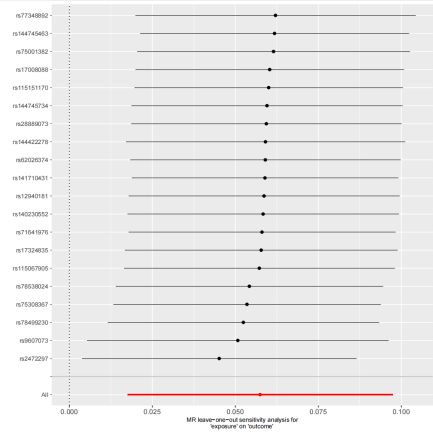 |
| Taurolithocholate 3-sulfate levels | Behenoyl dihydrosphingomyelin | 3-hydroxy-2-methylpyridine sulfate levels |
| 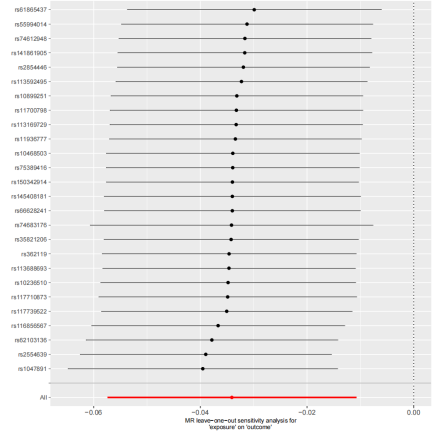 | 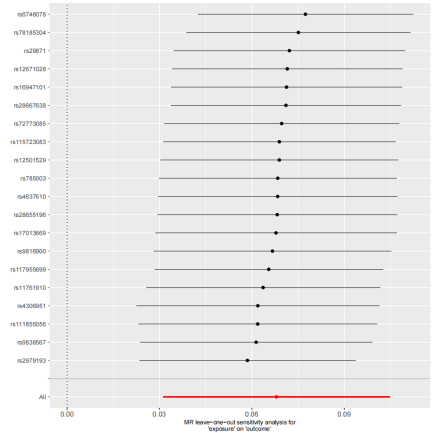 | 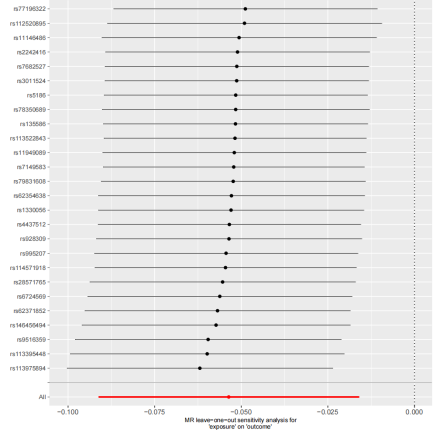 |
| ADP to glycine ratio | IMP to phosphate ratio | Glutamate to pyruvate ratio |
| 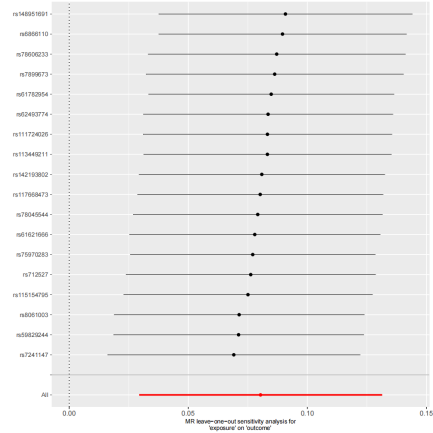 | 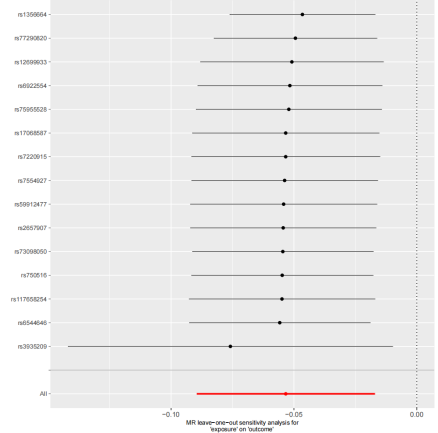 | 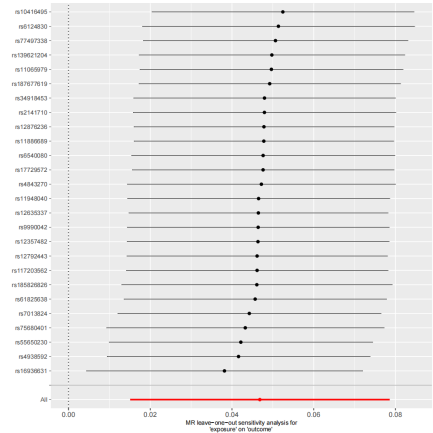 |
| Phosphate to glycerol ratio | 5-oxoproline to citrate ratio | Alpha-ketoglutarate to kynurenine ratio |
